# Magnetic Template Anion Polyacrylamide–Polydopamine-Fe_3_O_4_ Combined with Ultraviolet/H_2_O_2_ for the Rapid Enrichment and Degradation of Diclofenac Sodium from Aqueous Environment

**DOI:** 10.3390/polym12010072

**Published:** 2020-01-02

**Authors:** Qiang Sun, Huaili Zheng, Xuebin Hu, Jun Li, Rui Zhao, Chun Zhao, Wei Ding

**Affiliations:** 1 Key Laboratory of the Three Gorges Reservoir Region’s Eco-Environment, State Ministry of Education, Chongqing University, Chongqing 400045, China; xbhu@cqu.edu.cn (X.H.); ulquiorra_n@163.com (J.L.); 20171713027t@cqu.edu.cn (R.Z.); pureson@163.com (C.Z.); dingweidinghan@163.com (W.D.); 2 State Key Laboratory of Coal Mine Disaster Dynamics and Control, Chongqing University, Chongqing 400044, China

**Keywords:** diclofenac sodium, π–π stacking, charge neutralization, enrichment, degradation

## Abstract

In this study, a novel system was set up by preparing a magnetic flocculant combining with ultraviolet/H_2_O_2_ to realize the rapid enrichment and degradation of diclofenac sodium (DCFS). For the magnetic flocculant, template anion polyacrylamide (TAPAM) with anion micro-block structure was prepared. Thereafter, polydopamine was used to modify TAPAM, Fe_3_O_4_ nanoparticles was grafted to the modified TAPAM by chelation, named template anion polyacrylamide-polydopamine-Fe_3_O_4_ (TAPAM-PDA-Fe_3_O_4_). Furthermore, the TAPAM-PDA-Fe_3_O_4_ preparation protocol was optimized by the response surface method (RSM). In the DCFS enrichment section, the rapid separation of flocs from water was realized by an external magnetic field and it indicated that the π–π stacking effect was dominant in neutral/alkaline condition, whereas charge neutralization was favored in acidic conditions. Meanwhile, a DCFS enrichment kinetic curve was much fitted by the pseudo-second-order kinetic model and DCFS enrichment isothermal curve was close to the Freundlich isothermal model, indicating the dependence of DCFS quantity enriched by TAPAM-PDA-Fe_3_O_4_ and a multilayer heterogeneous enrichment process. The degradation experiment confirmed that DCFS was effectively degraded by ultraviolet/H_2_O_2_/TAPAM-PDA-Fe_3_O_4_ and the maximum value of DCFS degradation efficiency reached 98.1%. Furthermore, the regeneration experiment showed that the enrichment and degradation efficiency of DCFS could maintain a relatively high level in the initial three recycles.

## 1. Introduction

Diclofenac sodium (DCFS) represents a rising concern due to its ecotoxicological potential and current, sustained release into the aquatic environment. Furthermore, many regions and countries, such as Europe, America, China, and so on, have added DCFS to the priority substances monitoring list of water policy [1,2,3]. Approximately a hundred tons of DCFS are sold annually worldwide, about 15% of which is excreted unchanged by human race and is frequently detected in domestic wastewater [4]. In addition, the ever-increasing global population is expected to cause a sharp increase in the emissions of DCFS in the future. Several studies have shown that DCFS exposure demonstrates bioaccumulation in invertebrates, which has an ecotoxicological risk to the aquatic food chain [5]. At present, due to the limited biodegradability of sewage treatment plants, DCFS can’t be effectively dislodged by traditional sewage treatment methods [6,7,8,9].

Several technologies have been studied to reduce the DCFS concentration in aqueous environments, such as photolysis and electro-Fenton based treatments, to give some examples [10,11]. Nevertheless, the low concentration of DCFS in domestic sewage makes it hard to degrade the pharmaceutical effectively by using these methods directly [12]. It is suggested that DCFS degradation efficiency would be enhanced by realizing DCFS enrichment before degradation process. There are also several studies on the enrichment of DCFS from water, which provide the possibility to realize the combination of DCFS enrichment and degradation [13,14,15].

Anion polyacrylamide (APAM) synthesized by acrylamide and 2-acrylamido-2-methyl-1-propanesulfonic acid (AMPS) is a typical class of organic flocculant with high intrinsic viscosity and electronegative properties. The molecular chains of APAM could induce charge neutralization to remove cationic organics from wastewater [16]. 

Meanwhile, dopamine (DA), which is environment-friendly and energy-efficient, could undergo self-polymerization by ultraviolet (UV) radiation. So far polydopamine (PDA) has been applied in various areas, such as wastewater treatment, drug loading capsules and organic catalysts considering its biocompatible, good thermal stability and amazing adhesion on various materials since its first report by Messersith and his colleagues in 2007 [17,18,19]. It is interesting to note that polydopamine and DCFS are both rich in aromatic rings, which may lead to the π–π stacking effect between them [20].

Moreover, response surface methodology (RSM) is widely adopted in the area of parameter optimization, which includes Doehlert matrix, central composite designs, and Box-Behnken design (BBD) [21,22]. Among them, BBD is more efficient and it can be used by relatively few combinations of variables to determine the complex response function [23].

**Figure 1:**
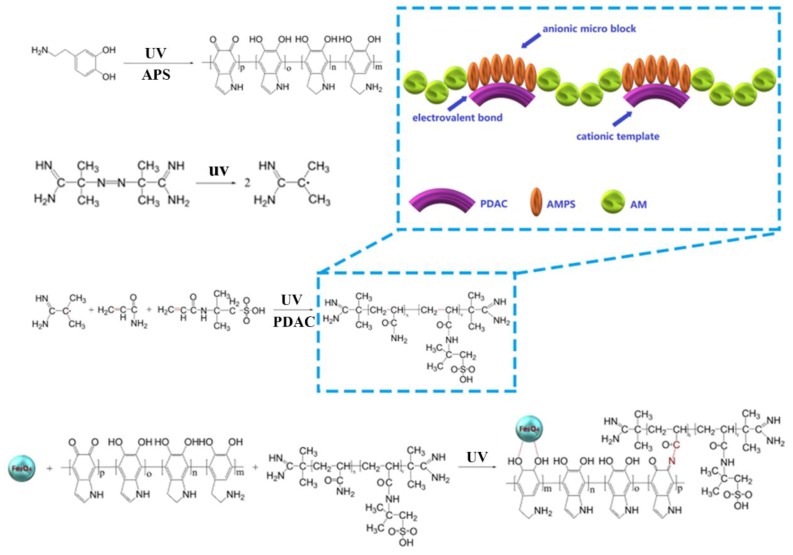
The scheme of preparing TAPAM-PDA-Fe_3_O_4_.

Given that, in this study, poly(acryloyloxyethyltrimethyl ammonium chloride) (PDAC) was adopted as a cationic template, and AMPS was used as the anion monomer to react with acrylamide by copolymerization under UV irradiation, so as to prepare the template anion polyacrylamide (TAPAM) with anion micro-block structure. After that, polydopamine was used to modify the TAPAM, where magnetic Fe_3_O_4_ nanoparticles was grafted to realize the rapid separation from the aqueous environment, and then the novel magnetic flocculant was prepared, named template anion polyacrylamide-polydopamine-Fe_3_O_4_ (TAPAM-PDA-Fe_3_O_4_) as shown in Figure 1. Moreover, RSM was employed with BBD to optimize the synthesis of TAPAM-PDA-Fe_3_O_4_, and the variables chosen for the design were pH, mole ratios (PDAC/AMPS, AMPS/AM, AM/DA, DA/Fe_3_O_4_) and irradiation time. The new magnetic flocculant was applied to the treatment of simulated wastewater containing DCFS, and then the rapid separation of flocs from water was realized by an external magnetic field. The separated mixture was re-immersed into a lower volume dilute sulfuric acid solution to increase DCFS concentration and to generate Fe^2+^ by grafted Fe_3_O_4_ nanoparticles. After adding the appropriate amount of H_2_O_2_ to the solution, it was settled in a UV irradiation environment. Finally, it was expected to realize the rapid enrichment, separation, and degradation of DCFS in order to overcome the related problems.

## 2. Materials and Methods

### 2.1. Materials

Acrylamide (AM) and acryloyloxyethyltrimethyl ammonium chloride (DAC) were purchased from Chongqing Lanjie Tap Water Company (Chongqing, China). Diclofenac sodium was obtained from Chengdu Kelong Chemical Reagent Corporation (Chengdu, China). 2-acrylamido-2-methyl-1-propanesulfonic acid (AMPS) was sourced from Shouguang Chemical Co. Ltd. (Shouguang, China). Photo-initiator 2,2′-azobis(2-methylpropionamide) dihydrochloride (V-50) was purchased from Ruihong biological technology Co. Ltd. (Shanghai, China). Dopamine hydrochloride ammonium persulfate was purchased from Aladdin Industrial Inc. (Shanghai, China). AM and AMPS were technical grade and the other reagents used in this study were analytical grade. All chemicals were used without further treatment and all solutions were prepared with ultrapure water (18 MΩ cm).

### 2.2. TAPAM-PDA-Fe_3_O_4_ Synthesis Optimization

The preparation of TAPAM-PDA-Fe_3_O_4_ described in Text S1 was optimized by Box-Behnken Design (BBD) and response surface method (RSM). Six factors: pH (X_1_), mole ratio of PDAC/AMPS (X_2_), AMPS/AM (X_3_), AM/DA (X_4_), DA/Fe_3_O_4_ (X_5_) and irradiation time (X_6_) were selected as the independent variables. Intrinsic viscosity was chosen as the response variable. As shown in Table 1 and Supplementary Table S1, B_54_ (3^6^) was used to improve the experiment and Supplementary Equation (S1) was used to predict the optimal condition.

### 2.3. Analytical Methods

FT-IR spectra were measured by a spotlight 200 FT-IR spectrometer (Nicolet IS10, Thermo Fisher Scientific, Waltham, MA, USA). X-ray photoelectron spectroscopy spectra was detected by the ESCALAB250Xi XPS spectrometer (Thermo Fisher Scientific, Waltham, MA, USA). Chemical constituents and thermal stability of the products were analyzed by a DTG-60H synchronal thermal analyzer (SHIMADZU, Kyoto, Japan). The morphology and fractal dimension of magnetic flocculant were detected by the high-resolution field emission scanning electron microscope (SEM, Quattro S, Thermo Fisher Scientific, Waltham, MA, USA) coupled with Image-Pro software (plus 6.0, Media Cybernetics, Rockville, MA, USA). The magnetic properties and crystal phases of magnetic flocculant were analyzed by a vibrating sample magnetometer (PPMS DynaCool 9, Quantum Design, San Diego, CA, USA) and a X-ray diffractometer (7000, Shimadzu, Kyoto, Japan), respectively. A Nano ZS90Zetasizer (Malvern Instruments Co. Ltd, Malvern, UK) was used to analyze zeta potential of DCFS aqueous solution.

### 2.4. Enrichment Experiments

DCFS standard aqueous solution of 2.0 mg L^−1^ was prepared in the lab, and the corresponding predetermined concentration (0.1, 1.0, 1.5, 2.0 mg L^−1^) was accurately prepared by diluting standard aqueous solution with ultrapure water. Standard TAPAM–PDA-Fe_3_O_4_ suspension of 10 mg mL^−1^ was prepared by adding 0.1 g TAPAM–PDA-Fe_3_O_4_ into 10 mL deionized water and then 0.5, 1.0, 1.5, 2.0, 2.5 and 3.0 mL of that were separately added into 250 mL DCFS solution of predetermined concentration. A program-controlled ZR4-6 jar test apparatus (Shenzhen Zhongrun Water Industry Technology and Development Co. Ltd, Shenzhen, China) was used to carry out enrichment experiment at ambient temperature and the mixture was stirred vigorously (300 rpm) for 1 min, then was stirred moderately (160 rpm) for 4 min, and finally stirred slowly (40 rpm) for 5 min. The rapid separation of magnetic flocculant from aqueous environment was achieved by settling a circular magnet under the beaker and the sample was extracted from 1 cm under the aqueous solution surface to detect the remained concentration by HPLC-UV (WATERS, Milford, MA, USA) combined with the COSMOSIL 3C_18_-MS-II column (Nacalai Tesque, Inc., Kyoto, Japan) at 276-nm detection wavelength. A mixture of acetic acid solution (3.0%)/acetonitrile (80:20) was used as the mobile phase, the flow rate and the largest injection volume of which were 1.0 mL min^−1^ and 50 μL, respectively. All results of DCFS remained concentration were averaged with three averaged measurements and the scale bar was obtained by calculating the standard deviation of the three values. The removal efficiency is described by Equation (1):

(1) $$\mathrm{Removal}\;\mathrm{efficiency}=\left(1-\frac{C_{f}}{C_{i}}\right)\times 100\%$$

where *C*_i_ and *C*_f_ are for the initial and the final concentration of DCFS, respectively.

### 2.5. Degradation Experiments

The separated magnetic flocculant prepared in Section 2.4 was then added into the 25 mL quartz conical flask with 10 mL dilute sulfuric acid solution (0.1 M) to generate Fe^2+^ by grafted Fe_3_O_4_ nanoparticles, and the pH was adjusted to 4.5. The concentration of Fe^2+^ in the sample after 60 min settlement was measured by the phenanthroline-colorimetric method. Then, a predetermined amount of H_2_O_2_ (n (Fe^2+^):n (H_2_O_2_) = 1:4) was injected into the solution. Meanwhile, the DCFS solution treated directly with Fe_3_O_4_/H_2_O_2_/sulfuric acid was used as control. The concentration of Fe^2+^, H_2_O_2_ and sulfuric acid in the control group was equal to that in the experimental group. The samples were both irradiated under UV environment at 365 nm wavelength by a GY-500 high-pressure mercury lamp for 60 min. The remaining amount of DCFS before and after irradiation was also detected by HPLC-UV (WATERS, Milford, MA, USA) and the degradation efficiency was calculated by Equation (1). The degradation products of DCFS were identified by ultraperformance liquid chromatography coupled with Vion ion mobility separation quadrupole time-of-flight tandem mass spectrometry (UPLC-Vion IMS Q-TOF-MS, ACQUITY I class, Waters, Milford, MA, USA).

### 2.6. Stability and Regeneration of TAPAM-PDA-Fe_3_O_4_

Stability and regeneration experiments were carried out to investigate the potential of TAPAM-PDA-Fe_3_O_4_ reutilization. After enrichment and degradation of sections of DCFS, the magnetic flocculant was extracted by an external magnetic field and revised by ethanol for three times, and it was used to repeat experiments in Section 2.4 and Section 2.5, and processed for five cycles. The removal and degradation efficiency of DCFS was analyzed by the method mentioned above. Thereafter, the FT-IR spectra of used TAPAM-PDA-Fe_3_O_4_ were obtained and virgin TAPAM-PDA-Fe_3_O_4_ was used as control. Meanwhile, the Fe^2+^ concentration of the solution settled for 60 min of every cycle containing sulfuric acid/TAPAM-PDA-Fe_3_O_4_ was detected by the method cited in Section 2.5. Further, the original DCFS solution (equal amount of DCFS) of every cycle, which contained an equal mass of Fe_3_O_4_ and the same concentration of sulfuric acid was used as control.

## 3. Results and Discussion

### 3.1. TAPAM-PDA-Fe_3_O_4_ Synthesis Optimization

**Figure 2:**
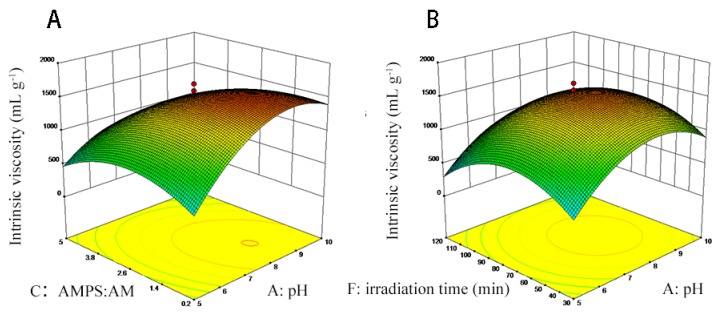
RSM analysis for the effect of pH coupled with mole ratio of AMPS/AM (**A**) and for the effect of pH combined with irradiation time (**B**).

The least square method shown in Supplementary Table S1 was used to measure the intrinsic viscosity regression model in consideration of the six factors. The regression model variance analysis and regression equation were presented in Supplementary Table S2 and Supplementary Equation (S2), respectively. As shown in Supplementary Table S2, the *p*-value was no more than 0.0001, suggesting statistical significance. Furthermore, X_1_, X_3_, X_6_, X_12_, X_32_, and X_62_ were significant model terms. The interaction effects of X_1_, X_3_ and X_1_, X_6_ on the intrinsic viscosity of TAPAM-PDA-Fe_3_O_4_ were depicted in Figure 2. The intrinsic viscosity TAPAM-PDA-Fe_3_O_4_ increased slightly with the increase of X_3_ (0.2–1.4) and then decreased as the X_3_ further increased (1.4–5.0) as shown in Figure 2A. On the contrary, the intrinsic viscosity increased significantly with the increase of X_1_ (5.0–8.5), then decreased slightly as the X_1_ further increased (8.5–10.0). It was more significant that pH showed on intrinsic viscosity of TAPAM-PDA-Fe_3_O_4_ than mole ratio of AMPS/AM as depicted in Figure 2A. The intrinsic viscosity of TAPAM-PDA-Fe_3_O_4_ strengthened sharply with the increase of X_6_ (30–75 min) and then decreased significantly as the X_6_ further increased (75–120 min) as presented in Figure 2B. The other factors (X_2_, X_4_, X_5_) had no significant effect on the intrinsic viscosity of TAPAM-PDA-Fe_3_O_4_ indicated from Supplementary Table S2. Furthermore, the simulated maximum value of by RSM was 1721.5 mL g^−1^ (X_1_ = 8.6, X_2_ = 0.50, X_3_ = 1.27 X_4_ = 1.48, X_5_ = 3.29, and X_6_ = 86.0 min). Moreover, six parallel TAPAM-PDA-Fe_3_O_4_ synthesis processes were carried out according to the theoretical optimal conditions. The actual intrinsic viscosity mean value was 1710.7 mL g^−1^, which was close to the simulated value, indicating the appositeness of the theoretical optimal condition.

### 3.2. Characterization of Magnetic Flocculant

#### 3.2.1. FT-IR and XPS Spectral Analysis

**Figure 3:**
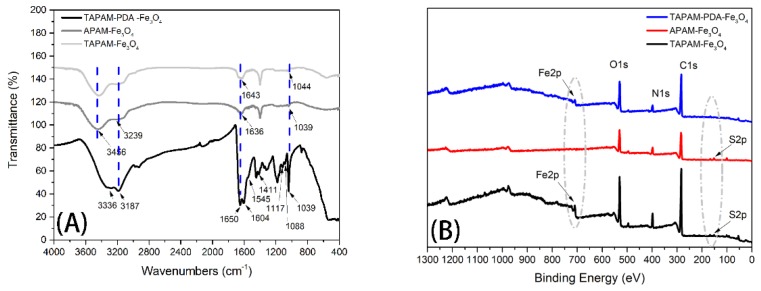
FT-IR (**A**) and XPS (**B**) spectra of TAPAM-PDA-Fe_3_O_4_, APAM-Fe_3_O_4_ and TAPAM-Fe_3_O_4_ (the dotted lines and dotted circles are corresponding to the peaks mentioned in Section 3.2.1).

Figure 3A illustrates the FT-IR spectra of TAPAM-PDA-Fe_3_O_4_, TAPAM-Fe_3_O_4_, and APAM-Fe_3_O_4_, respectively. As for TAPAM-PDA-Fe_3_O_4_, the characteristic peaks at 3336 cm^−1^ and 3187 cm^−1^ belong to the phenolic O–H and N–H stretching vibration, the peak at 1650 cm^−1^ was attributed to the aromatic ring stretching vibration and N–H bending vibration, the peak at 1545 cm^−1^ inclined the N–H shearing vibration, the peak at 1411 cm^−1^ was caused by phenolic C–O–H bending vibration, and the peak at 1117 cm^−1^ was due to C–O vibration, suggesting that PDA was grafted onto TAPAM successfully [24,25]. Furthermore, the peak at 1604 cm^−1^ was due to the C=O stretching vibration from AM and AMPS. Meanwhile the –SO_3_H– stretching bands from AMPS was found at 1088 and 1039 cm^−1^ [26,27]. As for APAM-Fe_3_O_4_ and TAPAM-Fe_3_O_4_, it was found that the C=O peak area at 1643 cm^−1^ in TAPAM-Fe_3_O_4_ was larger than that at 1636 cm^−1^ in APAM-Fe_3_O_4_. Furthermore, the wide scan XPS spectra of the TAPAM-PDA-Fe_3_O_4_, TAPAM-Fe_3_O_4_ and APAM-Fe_3_O_4_ was depicted in Figure 3B for analyzing the Fe_3_O_4_ nanoparticles distribution on the flocculants surface. The photoelectron lines at binding energies of 709.2 eV corresponding to Fe_2p_ were both found in curves of TAPAM-Fe_3_O_4_ and TAPAM-PDA-Fe_3_O_4_, but they didn’t exist in the curve of APAM-Fe_3_O_4_ (see dotted circles). As a result, PDAC used as the cationic template made the negative groups of AMPS more concentrated in the polymer chain and TAPAM grafted by PDA had abandon catechol groups, which may both contribute to make more Fe_3_O_4_ nanoparticles distribute on the flocculant surface and enhance the flocculant magnetic properties. Meanwhile, the binding energy of 166.3 eV related to S_2P_ was identified in the XPS spectra of APAM-Fe_3_O_4_ and TAPAM-Fe_3_O_4_, while that wasn’t recognized in TAPAM-PDA-Fe_3_O_4_ (see dotted circles). It was suggested that –SO_3_H– groups weren’t exposed on the surface when PDA grafted onto the TAPAM, which may lead to a multilayer heterogeneous enrichment process. Furthermore, compared with the C1s spectra of TAPAM-Fe_3_O_4_ and APAM-Fe_3_O_4_, the C1s spectra of TAPAM-PDA-Fe_3_O_4_ was deconvoluted into five components corresponding to C=C (283.7 eV), C–C (284.1 eV), C–N (286.0 eV), C–O (287.2 eV) and C=O (288.2 eV), which was depicted in Figure S1 [28,29]. It was further confirmed that abundant aromastic rings with hydroxyl groups were grafted on TAPAM.

#### 3.2.2. TGA-DSC Analysis

**Figure 4:**
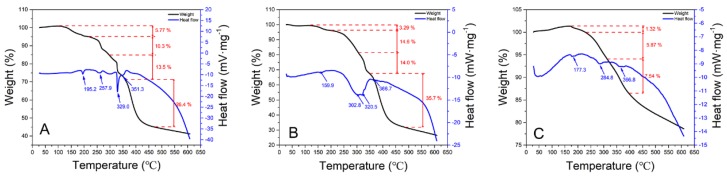
TGA-DSC analysis of (**A**) APAM-Fe_3_O_4_, (**B**) TAPAM-Fe_3_O_4_ and (**C**) TAPAM-PDA-Fe_3_O_4_.

Figure 4A–C show the Thermo Gravimetric and differential scanning calorimetry analysis (TGA-DSC) curves of APAM-Fe_3_O_4_, TAPAM-Fe_3_O_4_, and TAPAM-PDA-Fe_3_O_4_, respectively. It was observed in the TGA curves that four main stages for A and B and three main stages for C exist. The initial stages of the three TGA curves ranged 103.0–209.7 °C, 119.0–197.0 °C, and 164.5–229.6 °C with partial weight loss of about 5.77% for A, 3.29% for B, and 1.32% for C, owing to the confined water molecules bonded by hydrophilic groups in the molecular chains in the synthesis process, such as –SO_3_H– [30]. Thereafter, 10.3% weight loss (209.7–296.4 °C) for A and 14.6% weight loss (197.0–305.2 °C) for B were observed in the second stage. These results correspond to the thermal decomposition of –CO–NH– groups from A and B. The weight loss of 5.87% in the interval of 229.6–313.2 °C for C was corresponding to the thermal decomposition of aromatic rings and –CO–NH– groups [31,32]. In the third stage, 13.5% weight loss for A (296.4–357.1 °C), 14.0% weight loss for B (305.2–348.3 °C), and 7.54% weight loss for C (313.2–396.6 °C) were ascribed to the thermal decomposition of the –SO_3_H– groups [33]. As for the fourth stage of A and B, weight loss of about 26.4% and 35.7% correspond to the thermal decomposition of the copolymer backbone [34]. It was decomposed completely above 473.9 °C for A, above 485.4 °C for B, and above 608.9 °C for C. The corresponding residual weight percentage of 44.9%, 31.9% and 78.6% was corresponding to the residual Fe_3_O_4_ and carbon black, respectively [35]. The DSC curves of A and B both showed apparent four peaks of heat absorption appearing at 195.2 °C, 257.9 °C, 329.0 °C, 351.3 °C and at 159.9 °C, 302.8 °C, 320.5 °C, 366.7 °C, respectively. It was the derivative values of the corresponding TGA curves inflection points. Similarly, three heat absorption peaks appearing at 177.3, 284.8 and 366.3 °C for C were corresponding to the three inflection points of the TGA curve. These results demonstrated that APAM-Fe_3_O_4_, TAPAM-Fe_3_O_4_ and TAPAM-PDA-Fe_3_O_4_ had thermal stability between 27.9–209.7 °C, 28.1–197.0 °C and 29.4–229.6 °C, respectively.

#### 3.2.3. Morphological Analysis

**Figure 5:**
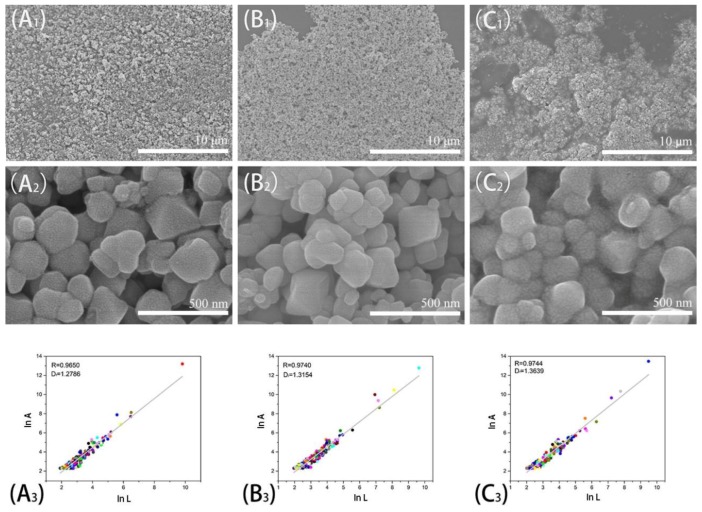
SEM and fractal dimensions of APAM-Fe_3_O_4_ (**A_1_**,**A_2_**,**A_3_**), TAPAM-Fe_3_O_4_ (**B_1_**,**B_2_**,**B_3_**) and TAPAM-PDA-Fe_3_O_4_ (**C_1_**,**C_2_**,**C_3_**). (The dots in A_3_, B_3_ and C_3_ were corresponding to the linear correlation of the logarithm of projected area A and the characteristic length L).

A vacuum freeze dryer was used to treat the samples to preserve the surface morphology of samples. The micro-structures of APAM-Fe_3_O_4_, TAPAM-Fe_3_O_4_ and TAPAM-PDA-Fe_3_O_4_ at a scale of 10 μm/500 nm were shown in Figure 5. The corresponding fractal dimensions of 1.2786, 1.3154, and 1.3639 are also shown in Figure 5, respectively. Interestingly, the specific surface area of APAM-Fe_3_O_4_, TAPAM-Fe_3_O_4_ and TAPAM-PDA-Fe_3_O_4_ increased gradually while the average particle size was reduced in sequence. The surfaces of the three magnetic flocculants were not smooth, which was caused by the self-assembly of polymer chains to form particles, and the particles are intertwined to form a porous network structure. It is favorable that enhancing the flocculation and bridging effect in the flocculation process. Furthermore, the morphology of APAM-Fe_3_O_4_, TAPAM-Fe_3_O_4_, and TAPAM-PDA-Fe_3_O_4_ changed gradually from fluffy to compact. This was caused by two reasons, on one hand, the template polymerization made the negative charge distribution more concentrated on the polymer chain so that the macromolecule backbone was more extended, which was favorable for the magnetic flocculant to form a lattice-like structure. On the other hand, the catechol groups from grafted PDA were strongly negative charged in the aqueous environment so that the internal charge repulsion of the molecular chain was increased, and the abundant aromatic rings from grafted PDA increased the inner-chain steric hindrance, which made the magnetic flocculant more spread in the aqueous environment. The lattice-like structure was further enhanced.

#### 3.2.4. XRD Patterns

**Figure 6:**
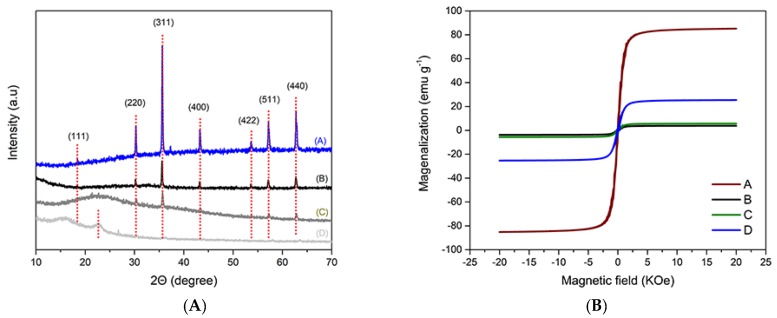
(**A**) XRD patterns and (**B**) magnetization curves of (A) Fe_3_O_4_, (B) APAM-Fe_3_O_4_ (C) TAPAM-Fe_3_O_4_ and (D) TAPAM-PDA-Fe_3_O_4_.

It was shown in Figure 6A that the XRD pattern curves of Fe_3_O_4_, TAPAM-PDA-Fe_3_O_4_, TAPAM-Fe_3_O_4_ and APAM-Fe_3_O_4_, respectively. The characteristic diffraction peaks of Fe_3_O_4_ in TAPAM-PDA-Fe_3_O_4_ and TAPAM-Fe_3_O_4_ were confirmed, indicating that the Fe_3_O_4_ crystal phase was unchanged after the polymerization section. However, there were not any characteristic peaks of Fe_3_O_4_ observed in APAM-Fe_3_O_4_, suggesting that the Fe_3_O_4_ nanoparticles were enmeshed into the polymer chain, which corresponds to the XPS analysis results. In addition, a broad peak at 22.7 °C, was observed, indicating the wrinkled surface structure of organic fraction.

#### 3.2.5. Magnetic Properties

As shown in Figure 6B, the saturation magnetization value was measured to be 25.3 emu g^−1^ for TAPAM-PDA-Fe_3_O_4_, 5.7 emu g^−1^ for TAPAM-Fe_3_O_4_, 3.8 emu g^−1^ for APAM-Fe_3_O_4_, and 85.1 emu g^-1^ for Fe_3_O_4_. The saturation magnetization value of TAPAM-PDA-Fe_3_O_4_ was relatively much higher than that of TAPAM-Fe_3_O_4_ or APAM-Fe_3_O_4_. It suggested that Fe_3_O_4_ nanoparticles chelated with grafted PDA was contributed to enhance the magnetization of flocculant. Although the saturation magnetization of TAPAM-PDA-Fe_3_O_4_ decreased compared with Fe_3_O_4_ particles, it could significantly facilitate the separation of flocs by an external magnetic field.

### 3.3. Enrichment Properties

#### 3.3.1. DCFS Initial Concentration Effect

**Figure 7:**
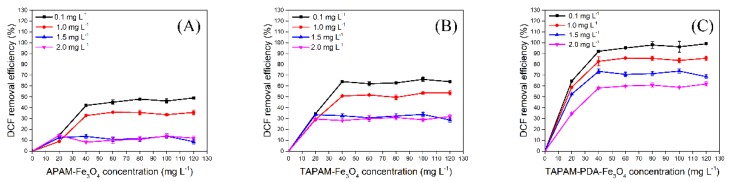
Effect of the DCFS initial concentration on enrichment properties ((**A**) for APAM-Fe_3_O_4_, (**B**) for TAPAM-Fe_3_O_4_, (**C**) for TAPAM-PDA-Fe_3_O_4_).

The enrichment capability of TAPAM-PDA-Fe_3_O_4_ for removing various concentration DCFS was investigated and APAM-Fe_3_O_4_ and TAPAM-Fe_3_O_4_ were used as control. For all three kinds of magnetic flocculants, the removal efficiency was higher as the DCFS initial concentration was 0.1 mg L^−1^ instead of 1.0, 1.5 and 2.0 mg L^−1^, as shown in Figure 7A–C. It was because that steric hindrance between DCFS and polymer chain was enhanced when DCFS initial concentration exceeded a certain value, which negatively affected the removal efficiency. It was also found that TAPAM-PDA-Fe_3_O_4_ had better DCFS removal efficiency than APAM-Fe_3_O_4_ or TAPAM-Fe_3_O_4_. The maximum removal efficiency of APAM-Fe_3_O_4_, TAPAM-Fe_3_O_4_ and TAPAM-PDA-Fe_3_O_4_ was 49.1%, 66.2%, and 99.1% as the DCFS concentration was 0.1 mg L^−1^, respectively. This phenomenon could be explained as follows. On one hand, the anion block structure made the negative charge more concentrated and strengthened the charge neutralization effect. On the other hand, the TAPAM modified by PDA had abandoned aromatic rings and absorbed DCFS through π–π stacking effect, which further improved the DCFS removal efficiency. As a result, the 0.1 mg L^−1^ DCFS was used in the following sections.

#### 3.3.2. DCFS Initial pH Effect

**Figure 8:**
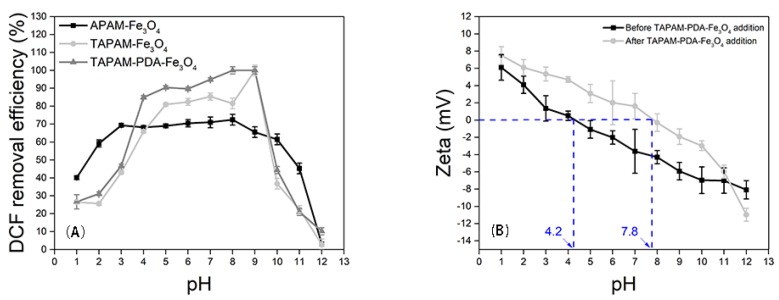
DCFS initial pH effect on the enrichment performance (**A**) and DCFS zeta potential at different pH before and after TAPAM-PDA-Fe_3_O_4_ injection (**B**).

The enrichment performance of APAM-Fe_3_O_4_, TAPAM-Fe_3_O_4_, and TAPAM-PDA-Fe_3_O_4_ was investigated under the conditions of various DCFS initial pH values. As seen in Figure 8A, in the range of pH 1.0–3.0, the DCFS removal efficiency was increased when the sample was treated by APAM-Fe_3_O_4_. In the interval of pH 3.0–8.0, the DCFS removal efficiency was maintained between 68.2%–72.4%. However, it decreased dramatically to 3.0% when the initial pH further increased to 12.0. As for TAPAM-Fe_3_O_4_ and TAPAM-PDA-Fe_3_O_4_, the removal efficiency curves of DCFS had the similar trend. In the range of pH 4.0–9.0, the enrichment performance of TAPAM-Fe_3_O_4_ and TAPAM-PDA-Fe_3_O_4_ was both better than that of APAM-Fe_3_O_4_. Furthermore, the enrichment performance of TAPAM-PDA-Fe_3_O_4_ was much better in the interval of pH 5.0–9.0, and DCFS removal efficiency was above 90.0% and the maximum value was above 95.0%. As a result, pH 8.0 was used in DCFS enrichment kinetics and isotherms analysis.

#### 3.3.3. Enrichment Mechanism Analysis

**Figure 9:**
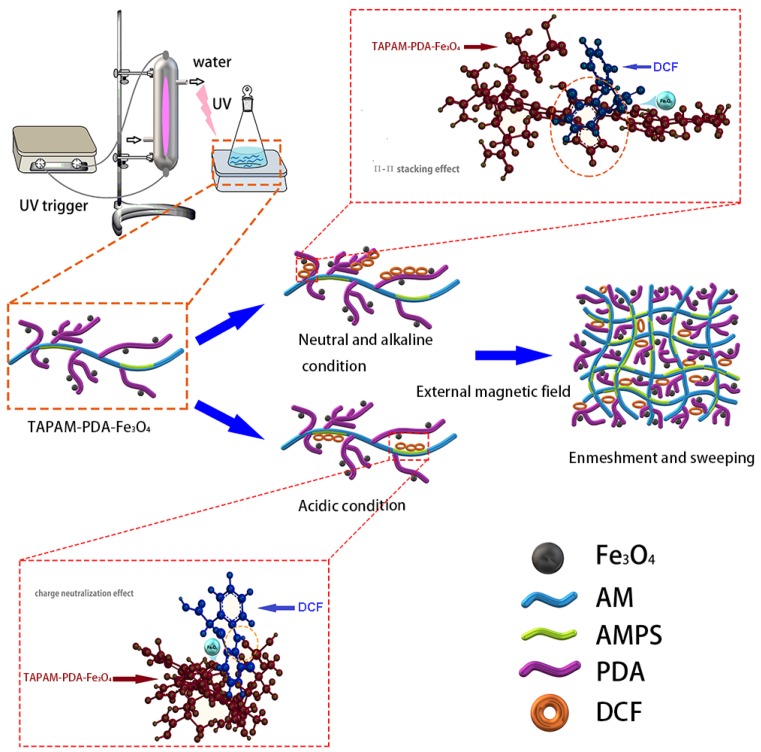
The scheme of DCFS enrichment mechanism by TAPAM-PDA-Fe_3_O_4._ (The detail schemes of the TAPAM-PDA-Fe_3_O_4_ structure, π–π stacking and charge neutralization effect were depicted in the dotted circles.)

The enrichment mechanism of TAPAM-PDA-Fe_3_O_4_ was further investigated. It was illustrated in Figure 8B that DCFS zeta potential obtained both before and after TAPAM-PDA-Fe_3_O_4_ treatment at different pH value. The DCFS isoelectric point was located at pH 4.2 before TAPAM-PDA-Fe_3_O_4_ treatment. However, the DCFS isoelectric point was around pH 7.8 after TAPAM-PDA-Fe_3_O_4_ addition, which was ascribed to the charge neutralization between –NH^+^ groups in DCFS and –SO_3_H– groups in TAPAM-PDA-Fe_3_O_4_. Furthermore, high DCFS maximum removal efficiency at pH 9.0 known from Figure 8A indicated the involvement of π–π stacking between DCFS and TAPAM-PDA-Fe_3_O_4_. At pH 10.0, the maximum removal efficiency reached 44.3%, which indicates that under relatively strong alkaline conditions, the electrostatic repulsion between the negatively charged groups also leads to a decrease in the π–π stacking efficiency, and the enmeshment/sweeping effect played a considerable role. In a nutshell, the whole process is depicted in Figure 9. First, DCFS was absorbed by abundant aromatic rings in TAPAM-PDA-Fe_3_O_4_ through π–π stacking in the interval of 7.0 < pH < 9.0. Second, it was charge neutralization between DCFS and –SO_3_H– groups in TAPAM-PDA-Fe_3_O_4_ that worked in the range of 1.0 < pH < 4.2. At last, large flocs were formed by the aggregation of small flocs through the external magnetic field.

#### 3.3.4. DCFS Enrichment Kinetics Analysis

**Figure 10:**
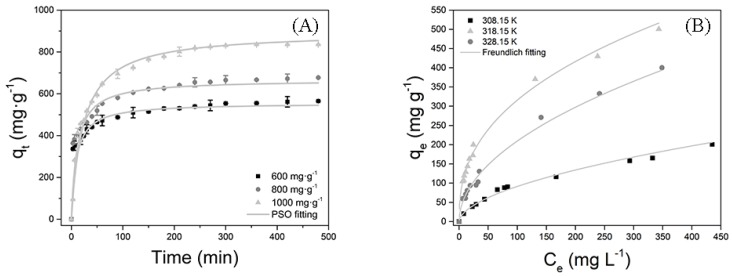
Enrichment kinetics of DCFS (**A**) and enrichment isotherms of DCFS (**B**) by TAPAM-PDA-Fe_3_O_4_ treatment.

The DCFS enrichment kinetics treated by TAPAM-PDA-Fe_3_O_4_ were investigated by pseudo-first order (PFO), pseudo-second order (PSO), and intraparticle diffusion models, which were expressed as Supplementary Equation (S3)–(S5). All the obtained data were simulated by the three kinetic models. As a result, the simulated parameters were depicted in Table 2. Compared with other two models, the PSO model correlation coefficients were much closer to 1.0, meanwhile the *q* value calculated by PSO model is much closer to the actual value. This confirmed that DCFS removal rate was dependent on the quantity of DCFS flocculated onto the surface of TAPAM-PDA-Fe_3_O_4_ [36,37]. The PSO model simulated curves of DCFS enrichment kinetics by TAPAM-PDA-Fe_3_O_4_ are presented in Figure 10A. The DCFS removal rate increased sharply in the first 30 min, but slowed down thereafter. The foremost DCFS rapid enrichment was due to the accessibility of aromatic rings and –SO_3_H– groups in TAPAM-PDA-Fe_3_O_4_. The succedent DCFS slow enrichment was due to the saturation of the flocculation sites.

#### 3.3.5. DCFS Enrichment Isotherms Analysis

The obtained experimental data were simulated by Dubinin-Radushkevich, Freundlich, and Langmuir isotherm models, which were expressed as Supplementary Equation (S6)–(S8). The final fitted parameters are all depicted in Table 3. It was conformed that Freundlich model correlation coefficients were much close to 1.0, suggesting that a multilayer heterogeneous flocculation of DCFS occurred onto the TAPAM-PDA-Fe_3_O_4_ [38,39]. In addition, the Freundlich isotherm simulated curves of DCFS at different temperatures were depicted in Figure 10B. The DCFS enrichment efficiency at 318.15 K was better than that at 308.15 K or 328.15 K. This indicated that the Brownian motion principle between DCFS and TAPAM-PDA-Fe_3_O_4_ had a great influence on the enrichment process. To some extent, it increased the collision possibility between DCFS and TAPAM-PDA-Fe_3_O_4_, which was positively contributed to DCFS enrichment. While beyond a certain limit, confined DCFS molecules by TAPAM-PDA-Fe_3_O_4_ tended to escape, which weakened DCFS enrichment process.

### 3.4. Degradation Performance

**Figure 11:**
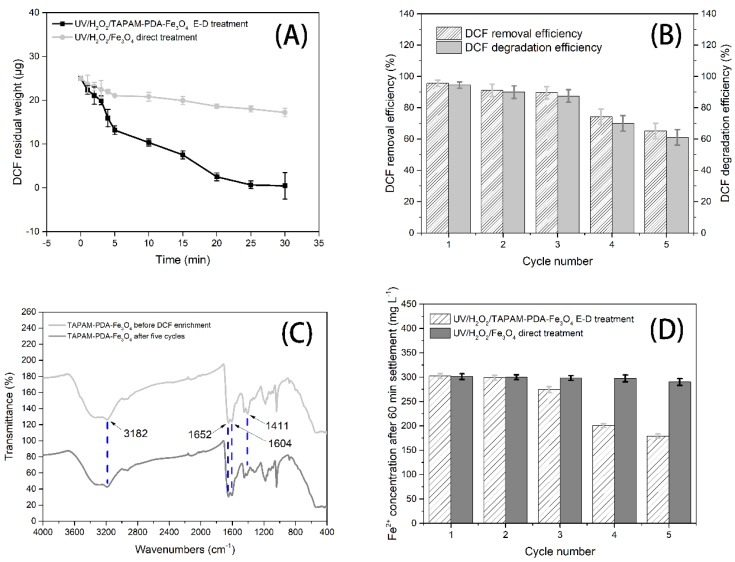
DCFS residual weight in the initial cycle (**A**), DCFS removal and degradation efficiency (**B**), FT-IR spectra of virgin and used TAPAM-PDA-Fe_3_O_4_ (**C**) and the concentration of Fe^2+^ in solution (**D**).

As shown in Figure 11A, the total amount of DCFS in the two samples separately was equal (25 μg) and when the DCFS simulated wastewater (0.1 mg L^−1^) was directly treated with UV/H_2_O_2_/Fe_3_O_4_, the degradation efficiency was low because of the low DCFS concentration. Meanwhile, the maximum degradation efficiency was 31.1% and the residual amount of DCFS was 17.2 μg after 30 min UV irradiation. However, the maximum degradation efficiency reached 98.1% and the DCFS residual amount was 0.48 μg when it was under UV/H_2_O_2_/TAPAM-PDA-Fe_3_O_4_ enrichment-degradation (E-D) treatment. This suggested that DCFS degradation could be realized efficiently when the enriched DCFS was re-dissolved into the dilute sulfuric acid solution (pH = 4.5) which was 0.04 times the original wastewater volume. Meanwhile, the degradation products of DCFS were identified by UPLC-Vion IMS QTOF-MS. Among the products, bis (6-methylheptyl) phthalate and 2, 6-dichloeoaniline were identified, except diclofenac sodium, which was depicted in Supplementary Figure S2. However, the degradation pathways of diclofenac sodium need further identification, and will be investigated in our next study. Moreover, various degradation processes of diclofenac sodium were summarized and compared in Table 4. It can be seen that the UV/H_2_O_2_/TAPAM-PDA-Fe_3_O_4_ process is a promising method to treat low concentration diclofenac sodium solution.

### 3.5. Stability and Regeneration of TAPAM-PDA-Fe_3_O_4_

In consideration of the stability of TAPAM-PDA-Fe_3_O_4_, it was reused for five cycles to enrich and degrade DCFS, and the FT-IR spectra of the revised magnetic flocculant are shown in Figure 11C. Compared with the initial TAPAM-PDA-Fe_3_O_4_, the characteristic peaks could be all found at 3182, 1652, and 1604 cm^−1^ in the revised magnetic flocculant. While the intensity of the characteristic peak at 1411 cm^−1^, which is ascribed to the phenolic C–O–H bending vibration, was weaker than the virgin TAPAM-PDA-Fe_3_O_4_ curve. This was due to the irreversible self-assembly of TAPAM-PDA-Fe_3_O_4_ and parts of the catechol groups were enmeshed inside the magnetic flocculant. However, the functional groups of TAPAM-PDA-Fe_3_O_4_ remained relatively stable. Meanwhile, the DCFS removal efficiency and DCFS degradation efficiency both remained in the relatively high value in the initial three circles, which were separately above 90.0% and 87.0%, as shown in Figure 11B. However, the removal and degradation efficiency in the fourth and fifth cycles degraded sharply, and the corresponding minimum values were 65.1% and 59.5%, respectively. The phenomena could be explained as follows. The decrease of DCFS removal efficiency was owing to the irreversible enmeshment of TAPAM-PDA-Fe_3_O_4_, which led to the decreasing amount of the active function groups on the surface of the magnetic flocculant. Furthermore, the decrease of DCFS degradation efficiency was caused by two aspects. On one hand, the decrease of DCFS removal efficiency would negatively affect the concentration of the concentrated DCFS solution. On the other hand, the amount of Fe_3_O_4_ nanoparticles grafted on the surface of TAPAM-PDA-Fe_3_O_4_ decreased through reacting with sulfuric acid, which led to the sequence decrease of Fe^2+^ concentration of DCFS solution in the fourth and fifth degradation cycles, as shown in Figure 11D. However, there was little effect on the initial three removal and degradation efficiency cycles.

## 4. Conclusions

In this study, TAPAM-PDA-Fe_3_O_4_ was successfully prepared to achieve a high-level enrichment of DCFS, up to 99.1%. Furthermore, the rapid separation of flocs from aqueous environment was realized by external magnetic field and higher maximum removal efficiency could be achieved with TAPAM-PDA-Fe_3_O_4_ (99.1%) instead of APAM-Fe_3_O_4_ (49.0%) or TAPAM-Fe_3_O_4_ (66.2%). In neutral/alkaline condition, it was π–π stacking that had a significant impact on flocculation performance. However, in acidic conditions, charge neutralization played a leading role. In the degradation section, it was found that the degradation efficiency of DCFS by UV/H_2_O_2_/TAPAM-PDA-Fe_3_O_4_ E–D treatment could reach 98.2%, compared with which was 31.1% of UV/H_2_O_2_/Fe_3_O_4_ direct treatment. Meanwhile, the TAPAM-PDA-Fe_3_O_4_ could be reused effectively for three circles to realize effective enrichment and degradation of DCFS. In summary, we have designed an integrated DCFS removal system, which can reach above 90.0% removal efficiency and above 87.5% degradation efficiency in the first three cycles. Looking forward, it is possible for the UV/H_2_O_2_/TAPAM-PDA-Fe_3_O_4_ system to treat wastewater containing DCFS effectively in relatively loose external conditions, thus holding great promise for further use in actual sewage treatment.

## Figures and Tables

**Table 1:** The independent variables levels designed in the B_54_ (3^6^).

Independent Variables	Code	Levels		
		−1	0	1
pH	X_1_	5	7.5	10
n_1_ (PDAC: AMPS)	X_2_	0.5	1.25	2
n_2_ (AMPS: AM)	X_3_	0.2	2.6	5
n_3_ (AM: DA)	X_4_	1	3.5	6
n_4_ (DA: Fe_3_O_4_)	X_5_	1	3.5	6
Irradiation time (min)	X_6_	30	75	120

**Table 2:** DCFS enrichment kinetic parameters at 318.15 K (initial pH 8.0).

Pseudo First-Order Kinetic Model					Pseudo Second-Order Kinetic Model			Intraparticle Diffusion Kinetic Model		
Initial Concentration (mg L^−1^)	*q*_max,exp_ (mg g^−1^)	*q*_e_ (mg g^−1^)	*k*_1_ × 10^2^ (g mg^−1^ min^−1^)	*R* ^2^	*q*_e_ (mg g^−1^)	*k*_2_ × 10^4^ (g mg^−1^ min^−1^)	*R* ^2^	*C* (mg g^−1^)	*k*_p_	*R* ^2^
600	561.7	197.5	0.91	0.9799	555.5	1.929	0.9991	351.9	11.57	0.8949
800	672.5	272.5	0.98	0.9887	666.6	1.364	0.9989	382.8	16.22	0.9021
1000	836.1	721.8	1.82	0.9426	909.1	0.5874	0.9986	317.4	30.04	0.8052

Where *q*_e_ and *q*_max,exp_ are the enrichment capacity of the magnetic flocculant at equilibrium and the actual maximum enrichment capacity of the magnetic flocculant, respectively. *k*_1_ and *k*_2_ are the rate constant of first-order and second-order flocculation, respectively. *k*_p_ is the intraparticle diffusion rate constant and *R*^2^ is the correlation coefficient.

**Table 3:** Isotherm parameters for DCFS enrichment treated by TAPAM-PDA-Fe_3_O_4_ at pH 8.0.

Langmuir Isotherm Model					Freundlich Isotherm Model			D-R Isotherm Model		
*T* (K)	*k*_L_ (L mg^−1^)	*q*_max_ (mg g^−1^)	*R*_L_^a^	*R* ^2^	*k*_F_	*n*	*R* ^2^	*q*_d_ (mg g^−1^)	*k*_d_ × 10^6^ (mol^2^ kJ^−2^)	*R* ^2^
298.15	0.02	144.9	0.048	0.965	7.15	1.81	0.994	124.7	1.74	0.748
308.15	0.044	366.3	0.022	0.946	50.4	2.51	0.997	361.6	1.22	0.862
318.15	0.025	266	0.038	0.935	20.5	1.98	0.998	261.8	1.9	0.806

*k*_L_ is for the Langmuir isotherm constant, *k*_F_ is corresponding to the Freundlich isotherm constant, *k*_d_ is the constant related to the mean free energy of flocculation and *R*_L_^a^ is the separation factor related to Langmuir model.

**Table 4:** Comparison of the diclofenac sodium degradation by different processes.

Processes	Water Matrix	Initial Concentration (mg L^−1^)	Degradation Conditions	Degradation Efficiency (%)	References
Photocatalysis	Aqueous solution	10	S-TiO_2_: 0.2–0.8 g L^−1^ pH: 6.0–11.0	93.0	[40]
Photocatalysis	Aqueous solution	5	TiO2: 4 g L^−1^	95.0	[41]
Photoelectrocatalysis	Aqueous solution	10	Persulate: 1–10 mM pH: 5.6–10.0	86.3	[42]
Photoelectrocatalysis	Aqueous solution	5	Pd/TNTs	67.7	[43]
Sonolysis	Aqueous solution	50–100	Ultrasonic frequency: 216–850 kHz	>90.0	[44]
UV/H_2_O_2_/TAPAM-PDA-Fe_3_O_4_	Aqueous solution	0.1	TAPAM-PDA-Fe_3_O_4_: 120 mg L^−1^ pH: 4.5	>90.0	This study

## Supplementary Materials

The following are available online at https://www.mdpi.com/2073-4360/12/1/72/s1. Text S1: Preparation of coagulant; Table S1: Six factors Box-Behnken design and the value of response function; Table S2: Variance analysis of regression model; Figure S1: C1s XPS spectra peak fitting of TAPAM-PDA-Fe_3_O_4_ (A), APAM-Fe_3_O_4_ (B) and TAPAM-Fe_3_O_4_ (C); Figure S2: The products ion spectra by UPLC-Vion IMS QTOF-MS.
