# Supplementary material for: Magnetic Template Anion Polyacrylamide–Polydopamine-Fe3O4 Combined with Ultraviolet/H2O2 for the Rapid Enrichment and Degradation of Diclofenac Sodium from Aqueous Environment
Source: Polymers (Basel). 2020 Jan 2;12(1):72. doi: 10.3390/polym12010072 (PMC7023637; doi:10.3390/polym12010072)
Supplement: Supplementary file 1 [file polymers-12-00072-s001.pdf]

polymers

Supplementary Material

Text S1 Preparation of coagulant

Preparation of PDA

The synthesis of PDA was carried out by oxidant-induced self-polymerization of dopamine. Typically, 2 g dopamine hydrochloride was dispersed in 50 mL ultrapure water adjusted to pH 7.5. After 5 min of sonication, the mixture was irradiated by ultraviolet light to initiate the polymerization reaction, which was allowed to proceed 1 hour. The precipitate was then retrieved by centrifugation and washed over three times with ultrapure water and ethanol.

Preparation of TAPAM

The synthesis of TAPAM was carried out in a 250 mL quartz jar. A 500-watt high-pressure mercury lamp was used as a UV light source for the polymerization process. A mixed aqueous solution composed of 7 g AM, 1g PDAC, 2 g AMPS was dissolved in 15 mL of ultrapure water. The initial pH of resulting solution was adjusted to 9.0 with 0.5 M NaOH or HCl. The predetermined amount of V−50 was added after the reaction solution was completely deoxygenated by bubbling with pure N_2 (99.99%) for 30 min. The reaction vessel was sealed immediately and exposed to radiation at room temperature for about 60 min. Then TAPAM was purified by ethanol several times. The white product was dried in a vacuum oven at 60 ℃ until constant weight.

Preparation of TAPAM-PDA-Fe_3O_4

Briefly, 2 g PDA, 2 g Fe_3O_4, 5 g TAPAM were dispersed in 100 mL ultrapure water, followed by N_2 purging for 5 min. The reaction vessel was sealed immediately and exposed to radiation at room temperature for about 60 min. Finally, the product was harvested by magnetic separation and washed repeatedly with ethanol. The Fe_3O_4-grafted polymer was obtained.

$$Y=\beta_0+\sum_{i=1}^{k}\beta_i X_i+\sum_{i=1}^{k}\beta_{ii} X_i^2+\sum_{i=1}^{k-1}\sum_{j=i+1}^{k}\beta_{ij} X_i X_j \quad \text{(Equation S1)}$$

Where β_0, β_i, β_{ii} and β_{ij} are regression coefficients, X_i and X_j are coded independent variables.

Equation S2:

Intrinsic viscosity = $1516.32+192.16X_1-36.04X_2-132.66X_3-85.93X_4-14.37X_5-23.92X_6+20.09X_1X_2-231.84X_1X_3-28.51X_1X_4+86.55X_1X_5-39.40X_1X_6+54.43X_2X_3+277.33X_2X_4+180.54X_2X_5-256.56X_2X_6+107.43X_3X_4-140.01X_3X_5+15.72X_3X_6-60.60X_4X_5-37.55X_4X_6-116.74X_5X_6-363.21X_1^2-102.96X_2^2-320.74X_3^2-271.17X_4^2-349.82X_5^2-506.54X_6^2$

$$q_t=q_e\times(1-e^{-k_1t}) \quad \text{(Equation S3)}$$

$$q_t=\frac{q_e^2k_2t}{q_ek_2t+1} \quad \text{(Equation S4)}$$

$$q_t=k_pt^{0.5}+C \quad \text{(Equation S5)}$$

Where q_e (mg g^{-1}) and q_t (mg g^{-1}) are the flocculation capacity of the magnetic flocculant at the equilibrium and at time t (min), respectively. k_1 and k_2 (g mg^{-1} min^{-1}) are the rate constant of first-order and second-order flocculation, respectively. k_p (mg g^{-1} min$^{-0.5}$) is the intraparticle diffusion rate constant, and C (mg g^{-1}) is also a constant.

$$ln\, q_e = ln\, q_d \text{-} K_d \varepsilon^2 \quad \text{(Equation S6)}$$

$$q_e = K_f C_e^{1/n} \quad \text{(Equation S7)}$$

$$q_e = \frac{q_m K_l C_e}{1+K_l C_e} \quad \text{(Equation S8)}$$

Where q_e (mg g^{-1}) and C_e (mg L^{-1}) are the flocculation capacity and concentration of DCF at equilibrium, respectively; q_m (mg g^{-1}) is the Langmuir constant related to the maximum flocculation capacity (mg g^{-1}) of the magnetic flocculant; and K_l (L mg^{-1}) is the Langmuir isotherm constant. K_f is the Freundlich isotherm constant, and n is the heterogeneity factor. q_d (mg g^{-1}) is the theoretical saturation capacity in the D-R model, K_d is the constant related to the mean free energy of flocculation, and ε is the Polanyi potential.

Table S1. Six factors Box-Behnken design and the value of response function.

Runs	X_1	X_2	X_3	X_4	X_5	X_6	Response value	
							Actual	Predicted
1	10	0.5	2.6	6	3.5	75	600.011	595.33
2	5	1.25	0.2	3.5	3.5	120	40.756	34.27
3	7.5	2	5	3.5	6	75	663.2	654.66
4	7.5	1.25	5	6	3.5	30	346.3	352.44
5	10	1.25	2.6	1	1	75	710.11	705.95
6	5	0.5	2.6	6	3.5	75	311.241	308.21
7	5	1.25	2.6	6	6	75	117.221	121.03
8	7.5	1.25	0.2	1	3.5	30	739.716	745.99
9	5	1.25	2.6	1	6	75	355.573	357.06
10	10	1.25	2.6	6	1	75	599.421	598.29
11	7.5	2	2.6	3.5	6	120	270.539	289.91
12	7.5	1.25	0.2	6	3.5	120	270.054	280.00
13	10	1.25	2.6	6	6	75	617.425	621.43
14	7.5	2	0.2	3.5	1	75	467.224	478.84
15	5	2	2.6	1	3.5	75	306.443	310.77
16	7.5	0.5	0.2	3.5	6	75	895.3	911.05
17	7.5	0.5	2.6	3.5	6	120	530.61	514.04
18	5	0.5	2.6	1	3.5	75	975.374	977.70
19	7.5	2	5	3.5	1	75	600.28	602.34
20	7.5	1.25	2.6	3.5	3.5	75	1515.18	1516.32
21	7.5	1.25	2.6	3.5	3.5	75	1517.11	1516.32
22	5	1.25	5	3.5	3.5	30	200.234	201.61
23	7.5	1.25	0.2	1	3.5	120	744.234	741.82
24	10	1.25	5	3.5	3.5	120	100.937	105.86
25	5	1.25	2.6	6	1	75	445.015	444.07
26	7.5	1.25	2.6	3.5	3.5	75	1516.408	1516.32
27	7.5	1.25	2.6	3.5	3.5	75	1516.408	1516.32
28	10	2	2.6	6	3.5	75	1120.78	1118.09

29	5	2	2.6	6	3.5	75	750.868	750.60
30	7.5	0.5	2.6	3.5	6	30	290.176	282.23
31	7.5	2	2.6	3.5	1	120	200.912	191.05
32	7.5	0.5	5	3.5	6	75	250.612	256.81
33	7.5	1.25	5	6	3.5	120	270.933	260.93
34	7.5	1.25	2.6	3.5	3.5	75	1516.408	1516.32
35	7.5	2	2.6	3.5	6	30	1083.06	1084.35
36	10	0.5	2.6	1	3.5	75	1378.25	1378.87
37	10	1.25	2.6	1	6	75	970.92	971.51
38	7.5	2	2.6	3.5	1	30	519.78	518.53
39	7.5	0.5	0.2	3.5	1	75	1030.14	1020.86
40	10	1.25	0.2	3.5	3.5	120	808.574	803.47
41	7.5	1.25	0.2	6	3.5	30	435.536	434.39
42	5	1.25	2.6	1	1	75	441.336	437.69
43	7.5	0.5	2.6	3.5	1	30	440.134	438.58
44	7.5	1.25	2.6	3.5	3.5	75	1516.408	1516.32
45	7.5	1.25	5	1	3.5	30	240.543	234.32
46	5	1.25	0.2	3.5	3.5	30	35.936	34.74
47	7.5	1.25	5	1	3.5	120	295.609	293.03
48	10	1.25	0.2	3.5	3.5	30	960.08	961.56
49	7.5	0.5	2.6	3.5	1	120	1120.81	1137.34
50	10	2	2.6	1	3.5	75	788.91	792.30
51	7.5	2	0.2	3.5	6	75	1110.63	1091.19
52	5	1.25	5	3.5	3.5	120	261.776	264.02
53	10	1.25	5	3.5	3.5	30	198.303	201.07
54	7.5	0.5	5	3.5	1	75	925.025	926.65

Table S2. Variance analysis of regression model.

Source	Sum of squares ×10^{-5}	df	Mean square ×10^{-5}	F value	P value prob＞F	
Model	102.886	27	3.811	3759.174	< 0.0001	Significance
X_1	8.863	1	8.863	8742.911	< 0.0001	
X_2	0.312	1	0.312	307.593	0.0014	
X_3	4.225	1	4.225	4168.215	< 0.0001	
X_4	1.772	1	1.772	1748.052	0.0085	
X_5	0.050	1	0.049	48.902	0.0172	
X_6	0.137	1	0.137	135.454	< 0.0001	
$X_1 X_2$	0.032	1	0.032	31.858	0.0097	
$X_1 X_3$	4.300	1	4.300	4242.002	0.0025	
$X_1 X_4$	0.130	1	0.130	128.303	0.4058	
$X_1 X_5$	0.599	1	0.599	591.134	0.1267	
$X_1 X_6$	0.124	1	0.124	122.539	0.0024	
$X_2 X_3$	0.237	1	0.237	233.799	0.0666	
$X_2 X_4$	6.153	1	6.153	6070.057	0.0086	
$X_2 X_5$	5.215	1	5.215	5144.777	0.0024	
$X_2 X_6$	5.266	1	5.266	5194.867	0.1221	
$X_3 X_4$	0.923	1	0.923	910.837	0.1063	
$X_3 X_5$	1.568	1	1.568	1547.000	0.0154	
$X_3 X_6$	0.040	1	0.039	39.007	0.056	
$X_4 X_5$	0.294	1	0.294	289.868	0.0014	
$X_4 X_6$	0.113	1	0.113	111.302	0.0085	
$X_5 X_6$	1.090	1	1.090	1075.486	0.1225	
X_1^2	13.569	1	13.570	13385.852	< 0.0001	
X_2^2	1.090	1	1.090	1075.626	0.0037	
X_3^2	10.582	1	10.580	10438.791	< 0.0001	
X_4^2	7.563	1	7.563	7461.210	0.0075	
X_5^2	12.587	1	12.590	12416.870	0.5677	
X_6^2	26.392	1	26.390	26035.461	< 0.0001	
Residual	0.026	26	1.014×10^{-3}		0.0026	
Lack of Fit	0.026	21	1.254×10^{-3}	320.796	0.3009	Not significance
Pure Error	1.955 ×10^{-5}	5	0.390×10^{-5}			
Cor Total	102.913	53				
